# Cognitive subgroups and the relationships with symptoms, psychosocial functioning and quality of life in first-episode non-affective psychosis: a cluster-analysis approach

**DOI:** 10.3389/fpsyt.2023.1203655

**Published:** 2023-07-27

**Authors:** Candice Tze Kwan Kam, Vivian Shi Cheng Fung, Wing Chung Chang, Christy Lai Ming Hui, Sherry Kit Wa Chan, Edwin Ho Ming Lee, Simon Sai Yu Lui, Eric Yu Hai Chen

**Affiliations:** ^1^Department of Psychiatry, School of Clinical Medicine, LKS Faculty of Medicine, The University of Hong Kong, Hong Kong SAR, China; ^2^State Key Laboratory of Brain and Cognitive Sciences, The University of Hong Kong, Hong Kong SAR, China

**Keywords:** cognitive heterogeneity, cognitive clusters, cognitive impairment, first-episode psychosis, functional outcome

## Abstract

**Introduction:**

Prior research examining cognitive heterogeneity in psychotic disorders primarily focused on chronic schizophrenia, with limited data on first-episode psychosis (FEP). We aimed to identify distinct cognitive subgroups in adult FEP patients using data-driven cluster-analytic approach, and examine relationships between cognitive subgroups and a comprehensive array of illness-related variables.

**Methods:**

Two-hundred-eighty-nine Chinese patients aged 26–55 years presenting with FEP to an early intervention program in Hong Kong were recruited. Assessments encompassing premorbid adjustment, illness-onset profile, symptom severity, psychosocial functioning, subjective quality-of-life, and a battery of cognitive tests were conducted. Hierarchical cluster-analysis was employed, optimized with k-means clustering and internally-validated by discriminant-functional analysis. Cognitive subgroup comparisons in illness-related variables, followed by multivariable multinominal-regression analyzes were performed to identify factors independently predictive of cluster membership.

**Results:**

Three clusters were identified including patients with globally-impaired (*n* = 101, 34.9%), intermediately-impaired (*n* = 112, 38.8%) and relatively-intact (*n* = 76, 26.3%) cognition (GIC, IIC and RIC subgroups) compared to demographically-matched healthy-controls’ performance (*n* = 50). GIC-subgroup was older, had lower educational attainment, greater positive, negative and disorganization symptom severity, poorer insight and quality-of-life than IIC- and RIC-subgroups, and higher antipsychotic-dose than RIC-subgroup. IIC-subgroup had lower education levels and more severe negative symptoms than RIC-subgroup, which had better psychosocial functioning than two cognitively-impaired subgroups. Educational attainment and disorganization symptoms were found to independently predict cluster membership.

**Discussion:**

Our results affirmed cognitive heterogeneity in FEP and identified three subgroups, which were differentially associated with demographic and illness-related variables. Further research should clarify longitudinal relationships of cognitive subgroups with clinical and functional outcomes in FEP.

## Introduction

Cognitive impairment is a core feature of schizophrenia and other psychotic disorders (1, 2). It is a major determinant of deterioration in functioning in everyday life including vocational functioning, independent living skills and social functioning (3–5). However, it is considered less recognizable and less manageable than positive symptoms of psychotic disorders as it cannot be improved effectively by antipsychotic treatment (1, 2). In fact, although early intervention service significantly improves functional outcome in patients with first-episode psychosis (FEP) (6), a substantial proportion of FEP patients still exhibit pronounced functional disability even in the presence of symptom remission (7–9). Hence, cognitive impairment constitutes an unmet therapeutic need in patients with psychotic disorders, particularly in relation to promoting early functional recovery.

An extant of literature has demonstrated deficits across multiple cognitive domains among patients with psychotic disorders relative to healthy controls, encompassing attention, processing speed, memory and executive functions (5, 10, 11). On the other hand, evidence has revealed cognitive heterogeneity in patients with psychotic disorders in terms of the severity and patterns of cognitive impairment (12). A growing body of research has utilized data-driven approach, e.g., cluster analysis, in an at attempt to identify homogeneous cognitive subgroups in psychotic disorders. Previous studies reported a 2- to 5-cluster solution on cognition, with the majority indicating three discrete cognitive subgroups characterized by patients with relatively-intact cognitive function, intermediate (i.e., moderately-severe deficits) and global cognitive impairment (i.e., widespread and severe deficits) (12–14). Some other studies also identified cognitive subgroups with more selective impairment in certain domains (12, 15, 16). Prior research has further explored differential associations of cognitive subtypes with clinical and functional characteristics of psychotic disorders. Although relatively mixed findings were observed across studies, accumulating data have suggested that a globally-impaired subgroup is generally associated with lower educational attainment, greater symptom severity (particularly negative symptoms) and worse psychosocial functioning compared with other cognitive subgroups (13, 14). Discrepant findings regarding the number, profiles and correlates of cognitive clusters derived might partly be attributable to cross-study methodological variations such as stages of illness (early vs. chronic or mixed) [e.g., (17)], clinical status (acute vs. clinically-stabilized), diagnostic categories included (non-affective psychoses only vs. both affective and non-affective psychoses) [e.g., (16, 18–20)], patient sample size, and adoption of different cognitive assessments, to name a few. Notably, the majority of earlier studies examining cognitive clusters focused on patients with chronic schizophrenia, which are confounded by clinical heterogeneity, illness chronicity and prolonged medication exposure. Until now, relatively few studies have applied data-driven approach to specifically delineate cognitive variability in FEP patients (18–22), with relatively modest sample size (ranged:105–204 patients, mostly with *n* < 150).

Better understanding and delineation of cognitive heterogeneity in the early course of psychotic disorders would facilitate elucidation of neurobiological mechanisms underlying various cognitive subtypes, and prediction of cognitive impairment trajectories, treatment response and illness outcome. To this end, we report a study conducted in a large representative cohort of Chinese adult patients presenting with first-episode non-affective psychosis to a specialized early intervention program with an aim to identify distinct cognitive subgroups using a cluster-analytic approach. In addition, we examined differential relationships of identified cognitive subgroups with a comprehensive array of illness-related variables encompassing premorbid adjustment, onset profile, various symptom domains, psychosocial functioning, and subjective quality of life (QoL). Based on prior literature in both FEP and chronic schizophrenia, we hypothesized that three cognitive subgroups would be identified by cluster analysis, including a relatively-intact, intermediately-impaired, and globally-impaired subgroups along a continuum of severity of cognitive impairment. We also anticipated that educational attainment, symptom severity and psychosocial functioning would be differentially associated with cognitive cluster membership.

## Materials and method

### Participants and setting

This study was conducted as part of the Jockey Club Early Psychosis (JCEP) Project (23), a territory-wide early intervention service which provided phasic-specific case management to adult individuals aged 26–55 years presenting with first-episode DSM-IV schizophrenia, schizophreniform disorder, schizoaffective disorder, brief psychotic disorder, delusional disorder, or psychotic disorder not otherwise specified (NOS) in Hong Kong. A total of 355 patients were recruited from publicly-funded generic adult psychiatric outpatient units. Patients with intellectual disability, neurological diseases and history of head injury that may compromise cognitive performance, substance-induced psychosis or psychotic disorder due to general medical condition were excluded. Data of this study were derived from baseline assessments (conducted with a mean of 119.7 days (median: 88 days) after treatment initiation) of a JCEP 4-year follow-up study, and baseline findings regarding depressive symptoms, duration of untreated psychosis (DUP), primary negative symptoms, and psychopathological network analysis have been reported elsewhere (24–27). The study was approved by local institutional review boards and written informed consent was obtained from all participants. Of the initial cohort, 289 patients who had completed all assessments including cognitive tests were retained as the study sample for the current report. Comparison between the study sample and the excluded participants (*n* = 66) revealed no significant differences in age at entry, gender and diagnostic categories. Excluded patients had significantly lower educational level than patients included in the current analysis (*p* < 0.01).

### Study assessments

Diagnostic ascertainment of each patient was based on reviewing all available information including Chinese-bilingual Structured Clinical Interview for DSM-IV (CB-SCID-I/P) (28) administered by senior research psychiatrists at intake, informant histories and medical records. Premorbid adjustment was evaluated using the Premorbid Adjustment Scale (PAS) (29). The overall PAS score encompassing developmental stages of childhood, early and late adolescence was derived according to the scoring method developed by Cannon-Spoor et al. (29). As in previous studies, we subdivided premorbid adjustment into social and academic functional domains (30, 31). An overall score for each of the two functional domains was computed by averaging the ratings of the relevant subscales across developmental stages (32). An overall premorbid adjustment score for each of the three developmental stages was also calculated by summing up all subscale scores and dividing by the maximum possible score. Interview for Retrospective Assessment of the Onset of Schizophrenia (IRAOS) (33) was employed to confirm the first-episode status and to determine age DUP and age at onset of psychosis. Positive and disorganization symptoms were assessed using the Positive and Negative Syndrome Scale (PANSS) (34) and were based on previous factor-analysis conducted in early psychosis sample (35). Negative symptoms were examined by the Scale for the Assessment of Negative Symptoms (SANS) (36). Calgary Depression Scale for Schizophrenia (CDSS) (37) was used to assess depressive symptoms. Insight was evaluated by PANSS G12 item score. Global psychosocial functioning was measured with the Social and Occupational Functioning Assessment Scale (SOFAS) (38). Subjective QoL was measured using a self-rated 12-Item Short Form Survey (SF12) (39). Data on treatment characteristics including use of second-generation antipsychotic and dose of antipsychotic medication (chlorpromazine equivalent doses were computed for analysis) (40) were obtained. A brief battery of cognitive assessments was administered comprising the following: a digital symbol subtest from the Wechsler Adult Intelligence Scale Revised (WAIS-R) (41) for processing speed; digit span from the WAIS-R for working memory; logical memory and visual reproduction subtests from the Wechsler Memory Scale Revised (WMS-R) (42) for verbal and visual memory, respectively; and category verbal fluency and Modified Wisconsin Card Sorting Test (MWCST) (43) for executive functioning. A group of healthy controls (*n* = 50), matched by age (mean = 36.4 years, SD = 12.7), gender (male: 30.0%) and educational level (mean = 10.3 years, SD = 1.9), was recruited in the community via advertisements. Controls were evaluated with the same battery of cognitive assessments as patients. Standardized z-score for each of the cognitive tests of individual patients was computed based on performance of healthy controls by subtracting the mean of controls’ score from each patient’s score and divided by the standard deviation of controls. All of the study assessments (other than diagnostic evaluation), including cognitive tests, were administered by research assistants who had received intensive training in the use of these assessments prior to participant recruitment.

### Statistical analysis

Hierarchical agglomerative cluster analysis (HCA) using squared Euclidean distance and Ward’s linkage method was performed to identify cognitive subgroups in FEP patients, based on the standardized z-scores of the six cognitive tests. Case similarity (i.e., distance between data points) was computed using squared Euclidean distance. Ward’s linkage method was applied as agglomeration procedure specification, and the distance between two clusters was defined by the increase of the sum of squares when merging them. The appropriate number of clusters was determined by collaborative inspection of the dendrogram and the agglomeration schedule coefficients in scree plot (as indicated by a sharp increase in the agglomeration coefficient). Then a k-means clustering (iterative partitioning) technique was applied to optimize the retained clusters, with initial partitions in the k-means solution defined using the cluster means derived from the hierarchical clustering procedure. A discriminant function analysis (DFA) was conducted to evaluate the internal validity of the cluster solution and to determine the predictive power of the cognitive performance in differentiating patients into discrete cognitive subgroups. Leave-one-out classification was used for assessing the reliability of the model generated by DFA. We then compared the identified cognitive subgroups on individual cognitive test scores, demographics, premorbid adjustment, onset profile, symptom domains, global functional status and subjective QoL, and treatment characteristics using a series of analysis of variance (ANOVAs), followed by post-hoc Turkey HSD test (with adjusted *p* < 0.01 indicating statistical significance) and chi-square tests as applicable. Those variables that were found to be statistically significant in preceding analyzes were also included in multivariate multinominal regression models to determine which factors independently predicted cognitive cluster membership. All analyzes were conducted using SPSS24.0 with significance level as *p* < 0.05, except post-hoc contrasts.

## Results

### Characteristics of the sample

Of the 289 participants in the study, 43.3% were male. The mean age of the sample was 38.2 years (SD = 8.3) and the median of DUP was 13 weeks (mean = 74.6, SD = 156.0). The majority (64.0%) were diagnosed with schizophrenia-spectrum disorder (schizophrenia: *n* = 134; schizophreniform disorder: *n* = 48; schizoaffective disorder: *n* = 3). For other non-affective psychoses, 12.8% (*n* = 37) of the cohort had brief psychotic disorder, 19% (*n* = 55) had delusional disorder and 4.2% (*n* = 12) had psychotic disorder NOS.

### Cluster analysis and cognitive profiles across clusters

**Figure 1:**
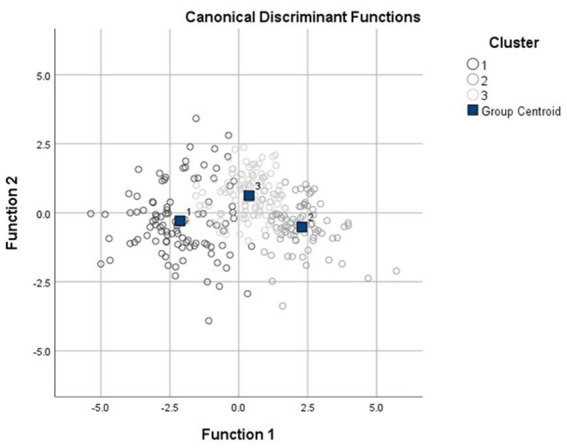
Discriminant plot of *k*-means three-cluster solution. The three clusters are (1) globally-impaired cluster, (2) intermediately-impaired cluster, and (3) relatively-intact clusters.

Inspection of the agglomeration scree plot and dendrogram revealed a three-cluster solution (Supplementary Figure S1). The discriminant plot of the final k-means cluster solution indicated relatively cohesive clusters with a concentration of cases around each of the three distinct centroids (Figure 1). The DFA yielded two discriminant functions which explained 92.4 and 7.6% of the variance, respectively (Wilks’ lambda = 0.197, *χ*^2^ (12) = 460.6, *p* < 0.001; Wilks’ lambda = 0.799, *χ*^2^ (5) = 63.68, *p* < 0.001), and the significant results indicated that the corresponding function explained the group membership well. The analysis also demonstrated that 86.5% of the cases were correctly classified in the respective group membership.

**Figure 2:**
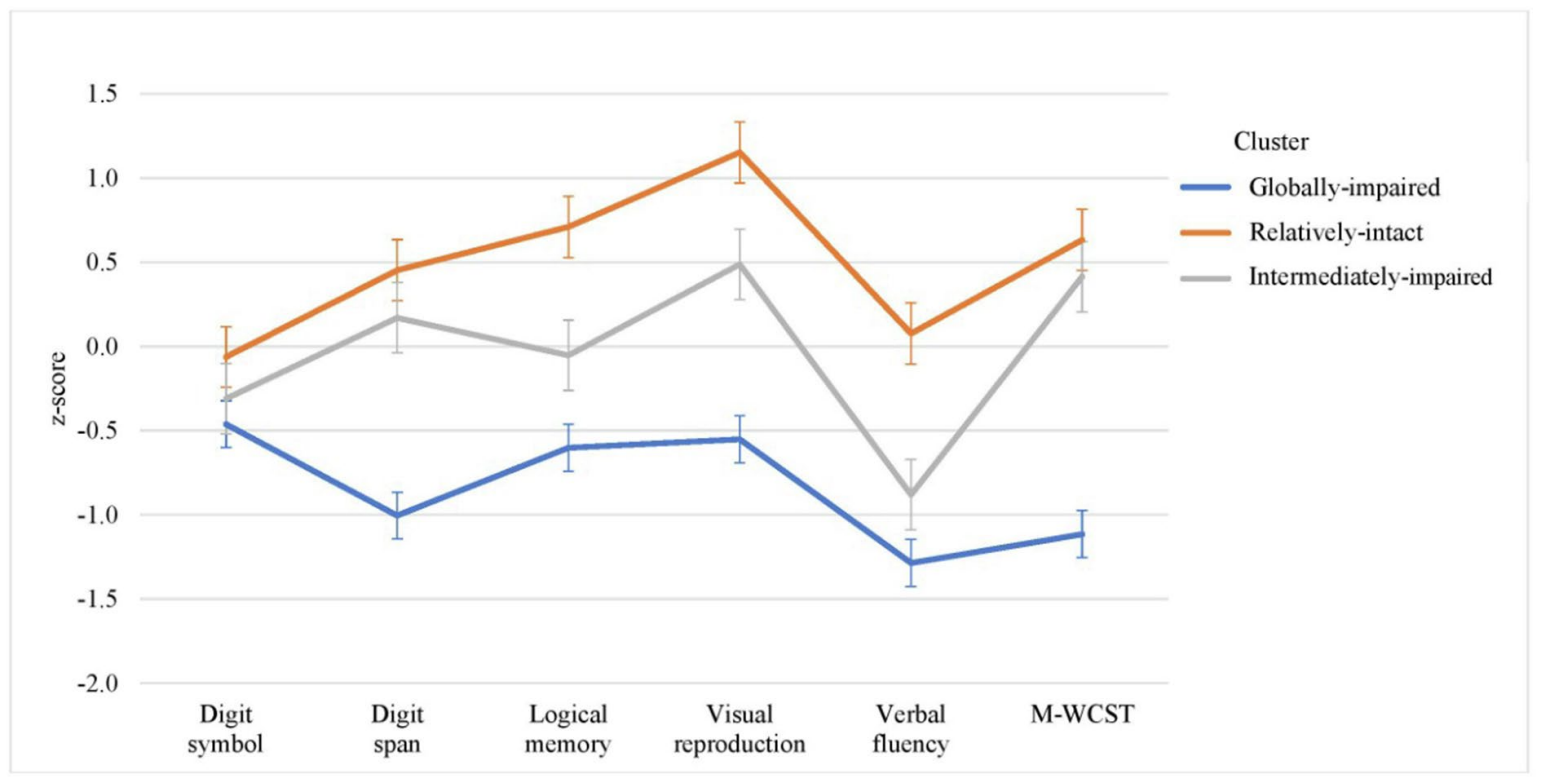
Cognitive performance among three cognitive clusters on each of the cognitive measures.

Cognitive profiles of three clusters are shown in Figure 2. Cluster 1 (*n* = 101, 34.9%) referred to globally-impaired cognitive (GIC) subgroup which displayed impairment in all of the six cognitive measures within 0.5–1.5 SD below the mean of controls’ performance, with more marked impairment in digit span, verbal fluency and MWCST (within 1.0–1.5 SD below the mean of controls). Cluster 2 (*n* = 112, 38.8%) was termed as intermediately-impaired cognitive (IIC) subgroup which exhibited mixed patterns of cognitive impairment including mild deficits in digit symbol and verbal fluency (i.e., within 1 SD below the mean of controls’ performance) and near-normal performance in the remaining cognitive measures (within 0.5 SD above the mean of controls). Cluster 3 (*n* = 76, 26.3%) referred to relatively-intact cognitive (RIC) subgroup which showed within 1 SD above the mean of controls’ performance in all cognitive measures. Table 1 summarizes the results of comparisons on the performance of individual cognitive measures across three clusters. There were significant differences in all of the six cognitive test scores between three cognitive subgroups. Post-hoc pairwise comparisons found that patients in GIC subgroup had significantly poorer performance than those in IIC and RIC subgroups in all cognitive measures. Patients in IIC subgroup significantly underperformed than those in RIC subgroup in digit symbol, logical memory, visual reproduction and verbal fluency. All of these between-cluster differences in cognitive test performance remained statistically significant after controlling for age at study entry and educational levels.

**Table 1:** Comparisons of cognitive performance among three cognitive subgroups in each of the cognitive measures.

Cognitive measures	Relatively-intact (*n* = 76)	Intermediately-impaired (*n* = 112)	Generally-impaired (*n* = 101)	F	*P*	Post-hoc comparison
Digit symbol	−0.06 (0.17)	−0.31 (0.13)	−0.46 (0.16)	152.20	<0.001	GIC < RIC GIC < IIC IIC < RIC
Digit span	0.45 (1.09)	0.17 (0.84)	−1.01 (0.97)	61.61	<0.001	GIC < RIC GIC < IIC
Logical memory	0.71 (0.83)	−0.05 (0.91)	−0.60 (0.91)	47.34	<0.001	GIC < RIC GIC < IIC IIC < RIC
Visual reproduction	1.15 (0.47)	0.49 (1.32)	−0.55 (1.61)	40.17	<0.001	GIC < RIC GIC < IIC IIC < RIC
Verbal fluency	0.08 (0.79)	−0.88 (0.76)	−1.29 (0.86)	64.33	<0.001	GIC < RIC GIC < IIC IIC < RIC
Modified Wisconsin Card Sorting Test	0.63 (0.52)	0.42 (0.71)	−1.12 (1.15)	118.95	<0.001	GIC < RIC GIC < IIC

GIC, globally-impaired cognitive subgroup; IIC, intermediately-impaired cognitive subgroup; RIC, relatively-intact cognitive subgroup.

### Subgroup comparisons on demographics, clinical and functional characteristics

As shown in Table 2, significant differences between three subgroups were observed in age at study entry, educational levels, age at onset, PANSS positive and disorganization symptom scores, SANS total scores, PANSS insight item scores, antipsychotic dose, SOFAS and SF12 total scores. There were no significant between-group differences in premorbid adjustment measures and DUP. Post-hoc pairwise comparisons revealed that patients in GIC subgroup were significantly older at entry and onset of psychosis, had fewer years of education, more severe positive, disorganization and negative symptoms, poorer insight and subjective QoL than those in IIC and RIC subgroups, and had higher antipsychotic dose than RIC subgroup. Patients in IIC subgroup had significantly fewer years of education and more severe negative symptoms than those in RIC subgroup. The RIC subgroup had significantly better global psychosocial functioning than both GIC and IIC subgroups. Multivariate multinominal regression analyzes (using GIC subgroup as a reference category) revealed that patients in GIC subgroup had significantly fewer years of education (*p* < 0.001) and greater disorganization symptom severity (*p* < 0.001) than those in RIC and IIC subgroups (Supplementary Table S1). Additionally, patients in GIC subgroup received higher dose of antipsychotic medication than those in RIC subgroup, with the group difference approaching statistical significance (*p* = 0.05).

**Table 2:** Comparisons among three cognitive subgroups in demographics, premorbid adjustment, clinical and functional characteristics.

Variables of interest	Relatively-intact (RIC) (*n* = 76)	Intermediately-impaired (IIC) (*n* = 112)	Globally-impaired (GIC) (*n* = 101)	*F/X^2^*	*P*	Post-hoc comparison
Demographics						
Male gender, *n* (%)	32 (42.11)	50 (44.64)	43 (42.57)	0.15	0.929	
Age at entry, mean (SD)	35.16 (7.76)	37.77 (8.02)	40.89 (8.13)	11.39	**<0.001**	GIC > IIC, GIC > RIC
Years of education, mean (SD)	13.28 (2.82)	11.36 (3.65)	9.08 (3.29)	35.32	**<0.001**	GIC < IIC < RIC
Premorbid and onset profiles						
Premorbid adjustment measures, mean (SD)						
PAS overall score	0.15 (0.15)	0.18 (0.16)	0.18 (0.17)	0.95	0.389	
PAS childhood score	0.17 (0.17)	0.14 (0.15)	0.16 (0.16)	0.46	0.630	
PAS early adolescence score	0.20 (0.19)	0.15 (0.15)	0.19 (0.17)	1.72	0.181	
PAS late adolescence score	0.18 (0.21)	0.16 (0.15)	0.17 (0.18)	0.37	0.690	
PAS academic domain score	0.21 (0.15)	0.15 (0.15)	0.19 (0.20)	0.42	0.655	
PAS social domain score	0.17 (0.21)	0.15 (0.18)	0.17 (0.18)	2.27	0.105	
Age at onset of psychosis, mean (SD)	33.47 (7.85)	36.13 (8.45)	39.09 (8.96)	9.65	**<0.001**	GIC > IIC, GIC > RIC
Log DUP, mean (SD)	1.90 (0.97)	1.85 (0.94)	2.07 (0.88)	1.67	0.190	
Diagnosis of schizophrenia-spectrum disorder,^a^ *n* (%)	46 (60.53)	71 (63.39)	68 (67.33)	0.90	0.637	
Symptom severity						
PANSS positive symptom score, mean (SD)	8.24 (3.99)	8.42 (3.26)	9.90 (4.60)	5.11	**0.007**	GIC > IIC, GIC > RIC
PANSS disorganization score, mean (SD)	7.46 (1.28)	8.19 (2.38)	9.77 (3.45)	18.86	**<0.001**	GIC > IIC, GIC > RIC
SANS total score, mean (SD)	3.03 (5.37)	5.62 (8.41)	8.77 (10.94)	9.53	**<0.001**	GIC > IIC > RIC
CDSS total score, mean (SD)	1.79 (3.25)	2.03 (3.62)	2.78 (3.68)	1.97	0.141	
Good insight,^b^ *n* (%)	63 (82.89)	94 (83.93)	69 (68.31)	4.69	**0.010**	GIC < IIC, GIC < RIC
Psychosocial functioning and subjective quality of life						
SOFAS score, mean (SD)	65.37 (13.97)	59.80 (12.72)	56.70 (11.94)	10.02	**<0.001**	GIC < RIC, IIC < RIC
SF12 total score, mean (SD)	132.89 (46.69)	132.83 (42.06)	115.07 (47.73)	5.03	**0.007**	GIC < IIC, GIC < RIC
Treatment characteristics						
Use of second-generation antipsychotics, *n* (%)	56 (73.7)	78 (69.6)	71 (70.30)	6.27	0.353	
Chlorpromazine equivalents, mg/day, mean (SD)	140.86 (131.40)	157.16 (113.76)	196.53 (164.43)	3.97	**0.020**	GIC > RIC

CDSS, Calgary Depression Scale for Schizophrenia; DUP, Duration of untreated psychosis; PANSS, Positive and Negative Syndrome Scale; PAS, Premorbid Adjustment Scale; SANS, Scale for the Assessment of Negative Symptoms; SD, Standard deviations; SF12, 12-Item Short Form Survey; SOFAS, Social Occupational Functioning Assessment Scale.

a Schizophrenia-spectrum disorder included schizophrenia, schizophreniform and schizoaffective disorder, while other non-affective psychoses included brief psychotic disorder, delusional disorder and psychosis not otherwise specified.

b Good insight was defined as PANSS G12 (Insight) item score ≤ 3.The blod values indicate *p* < 0.05, i.e., statistically significant.

## Discussion

To our knowledge, this is the largest study to examine cognitive heterogeneity in FEP patients using a data-driven cluster-analytic approach and to comprehensively assess differential relationships of cognitive subgroups with various illness-related characteristics. The current investigation is also the first of its kind conducted in non-Western regions and in the Chinese population. Two major findings emerged from the study. First, we identified three discrete cognitive subgroups, characterized by global impairment, intermediate impairment and relatively-intact cognitive functioning. Second, these cognitive clusters exhibited significant between-group differences in educational attainment, symptom severity, treatment characteristics, psychosocial functioning and subjective QoL.

Our finding of three-cluster solution concurs with the majority of previous studies which derived three distinct cognitive subgroups based on cluster analysis in both FEP (19, 20, 22) and chronic schizophrenia samples (14). Specifically, we found that patients classified as RIC subgroup accounted for 26.3% of our FEP sample, which is consistent with a recent systematic review showing that one-fourth of patients with schizophrenia-spectrum disorder displayed relatively-preserved cognitive functioning compared to healthy controls (14). For the two cognitively-impaired subgroups, 34.9 and 38.8% of patients were categorized as GIC and IIC subgroup, respectively. Patients in GIC subgroup showed deficits across all cognitive tests with 0.5–1.5 SD below the mean of healthy participants’ performance, whereas those in IIC subgroup displayed mixed pattern of cognitive dysfunction which comprised mild degree of deficits in digit symbol and verbal fluency as well as near-normal performance in the other cognitive measures. Our results thus affirm cognitive heterogeneity in first-episode population. Of note, our cognitively-impaired subgroups had comparatively milder degree of cognitive deficits than those with chronic schizophrenia. Prior cluster-analysis research on chronically-ill samples generally found that patients classified as having global cognitive impairment were characterized by widespread and more severe deficits of >1.5 SD below healthy control comparison (14). Conversely, most first-episode studies reported less severe overall cognitive deficits. For instance, Uren et al. (19) found 27.8% FEP patients with preserved cognitive functioning and 54.9% with “moderate cognitive impairment” of <0.5 SD below the mean of healthy controls, while Amoretti et al. (21) and Wenzel et al. (44) classified a large proportion of patients with FEP (43.9%) and recent-onset psychosis (62%) as having relatively-intact cognitive functioning, respectively.

Upon examining the patterns of cognitive profile of individual cognitive clusters, we found that the GIC subgroup displayed more pronounced deficits in verbal fluency, MWCST and digit span compared with the other cognitive tests. This finding thus suggested that, among various cognitive domains, executive functioning and working memory (albeit to a lesser extent) were relatively more impaired in patients with GIC subgroup. It is noted that some previous research has also conceptualized working memory as one of the separable cognitive components subsumed under executive functioning (45). Executive dysfunction, particularly impaired switching and flexibility, has been found to predict poor vocational outcome in FEP patients (46). One recent study even indicated that executive functioning performance specifically delineated the two clusters of chronic schizophrenia patients with intermediate cognitive impairment (47). Given that executive functioning comprises multiple individual cognitive processes, which were not comprehensively assessed in the current study, further research adopting a fractionated approach in examining executive functioning (45, 48) would facilitate clarification of whether there is any potential selective association of executive functioning profile with cognitive cluster membership in FEP patients. Nevertheless, in line with earlier cognitive-cluster studies in first-episode samples (19, 20, 22), our results indicate that cognitive cluster membership was primarily based on quantitative rather than qualitative difference in cognitive performance (with most of the cognitive tests showing graded pattern of impairment, i.e., RIC < IIC < GIC), thereby suggesting that cognitive impairment in FEP may represent a continuum of severity instead of the presence of distinct, domain-specific subtypes of the disorder. Owing to the relative paucity of existing data on cognitive subgrouping in FEP patients, further investigation is required to delineate cognitive variability in the early stage of illness.

Our results noted that years of education significantly decreased with increasing severity of impairment across cognitive subgroups (i.e., GIC < IIC < RIC). This is in line with past research on both chronic schizophrenia and early psychosis showing that patients with relatively-intact cognitive functioning had higher educational attainment than those in cognitively-impaired clusters (14, 17, 19, 22, 44). Substantial body of research also recognized close association between educational attainment and cognitive abilities across a lifespan (49). Contrary to a recent study revealing that relatively-intact subgroup displayed better premorbid scholastic performance than cognitively-impaired counterparts with first-episode schizophrenia (22), we failed to observe any significant differential associations between cognitive subgroups and various measures of premorbid adjustment. It should be noted that premorbid adjustment has rarely been investigated in cognitive cluster-analytic research on psychotic disorders. Nonetheless, evidence has indicated that poorer premorbid adjustment is related to worse cognitive impairment in psychotic disorders, particularly premorbid academic functioning (32, 50). Previous research examining cognitive developmental trajectories before onset of schizophrenia has also demonstrated that patients with long-term compromised premorbid cognition (with low premorbid and current intelligence) had significantly lower educational attainment than patients exhibiting cognitively-stable trajectory with normative premorbid intelligence (51, 52). Thus, the consistency between premorbid adjustment/intelligence and educational attainment regarding their relationship with cognitive impairment demonstrated in some past studies on psychotic disorders was not evident in our analyzes. Of note, our negative finding might partly be attributable to the nature of our FEP sample comprising only adult patients aged 26–55 years (i.e., over-represented by adult-onset psychosis), which contrasts with those earlier first-episode studies that also included patients at younger age or focused solely on adolescent and young adult patients (e.g., 15–25 years) (19, 22). Given that young age at onset (especially adolescent-onset) is in general associated with poorer premorbid adjustment relative to older age at onset, our sample might had comparatively lower degree of and less variance in premorbid functional impairment, thereby obscuring its potentially significant yet subtler association with cognitive subgroups. Moreover, premorbid functional assessment in our relatively older-aged sample may be more susceptible to recall bias, compared with younger-onset patients, due to a more prolonged duration between premorbid stage and illness onset, which in turn may result in less accurate evaluation of premorbid adjustment. Alternatively, most prior studies reported no association between age and cognitive clusters in FEP patients (18–20, 22), while one first-episode study found that later age at onset was linked to relatively-intact subgroup relative to the cognitively-impaired subgroup (21). Our result that “younger” age at onset was significantly related to GIC subgroup relative to IIC and RIC subgroups contrasts to the aforementioned findings, but should be treated with caution owing to an older age range of our sample compared with previous first-episode studies. Results of our multivariable multinominal regression analyzes also indicated that age or age at onset was not independently predictive of cognitive cluster membership.

We found that patients in GIC subgroup had significantly more severe positive and disorganization symptoms and poorer insight than counterparts in IIC and RIC subgroups. In particular, negative symptoms were differentially related to cognitive cluster membership, with symptom levels increased with patient subgroups of increasing severity of cognitive impairment (i.e., GIC > IIC > RIC). Our results thus accord with most previous studies showing that severely-impaired cognitive cluster experienced the greatest overall symptom severity, especially negative symptoms (14, 17–21). Recent data have further suggested that cognitive cluster membership at baseline was associated with negative symptom severity at 6- to 12-month follow-up in FEP patients (19, 20). These findings echo with a large body of evidence demonstrating significant associations between negative symptoms and cognitive deficits in both chronic and early course of illness (48, 53, 54), with accumulating data further indicating that baseline cognitive dysfunction predicts subsequent development of early-stage persistent negative symptoms in first-episode patients (55, 56). It is posited that cognitive impairment could affect the manifestations of negative symptoms as more preserved cognitive function is essential for individuals’ ability to plan, initiate and execute goal-directed behaviors. Alternatively, diminished motivation (or termed amotivation), a core subdomain of negative symptoms, was found to adversely influence cognitive performance in schizophrenia patients (57). Accumulating evidence demonstrated that schizophrenia patients exhibited effort-based decision-making impairment, with reduced willingness to expend effort for reward being associated with more severe amotivation (58). Our recent report further indicated significant association between decreased “cognitive” effort expenditure and higher levels of amotivation in FEP patients (59). Thus, amotivation and poor effort of patients may moderate and compromise their cognitive performance.

Consistent with the literature on cognitive subgrouping in psychotic disorders (14, 17–22), our results noted that the two cognitively-impaired subgroups exhibited significantly lower levels of psychosocial functioning than RIC subgroup. This is in agreement with substantial evidence showing that cognitive impairment is critically linked to poor functional outcome in early psychosis (48, 60–62). Furthermore, our study is the first to demonstrate differential relationship between cognitive clusters and subjective QoL in FEP, with RIC subgroup having significantly better subjective QoL than the two cognitively-impaired subgroups. Although existing data mostly found lack of significant association between subjective QoL and cognitive deficits in psychotic disorders (63), recent studies using structural equation modeling approach revealed that cognitive dysfunction was indirectly linked to subjective QoL via the mediation of psychosocial functioning (64, 65). Notably, our multinominal regression analysis showed that the difference between GIC and RIC subgroups on receipt of antipsychotic dose (with higher CPZ dose in GIC subgroup relative to RIC subgroup) approached statistical significance suggested that antipsychotic treatment may affect and potentially confound the study results. Previous research revealed that antipsychotics, particularly at high dose, may have negative effect on cognitive performance in schizophrenia patients (66). Evidence also observed that such negative effect varies with individual antipsychotics and specific cognitive domains (67). The finding of higher CPZ dose in GIC-subgroup patients might, on the other hand, reflect the need for increased intensity of antipsychotic treatment for their greater symptom severity, relative to those in RIC subgroup. Future investigation in medication-naïve FEP patients may help differentiate the effect of antipsychotics and illness on cognitive clustering and subgroup comparison. Taken together, our cluster-analysis results of three distinct cognitive subgroups in FEP patients were empirically supported by their significant differential associations with educational attainment, symptom severity, psychosocial functioning and subjective QoL. Multivariate multinominal regression analyzes, which took into consideration various significant variables, further showed that (fewer) years of education and (greater) severity of disorganization symptoms significantly delineated patients in GIC subgroup from those in RIC and IIC subgroups.

The study has several methodological limitations. First, the cross-sectional study design precludes us from establishing the causality between cognitive cluster membership and illness-related variables. Prospective research is warranted to clarify the longitudinal relationships of cognitive subgroups with clinical and functional outcomes in FEP. Second, we used a relatively brief battery of cognitive assessments which may not adequately capture the breadth and degree of impairment across multiple cognitive domains. Moreover, social cognition, which was found to be impaired in first-episode populations (68), was not evaluated in the study. Third, our finding that a relatively large proportion of our patients were categorized as relatively-intact or intermediately-impaired may indicate possible selection bias. Results of attrition analysis that patients retained in the current analysis had higher educational attainment than the excluded participants also suggest that our study sample may have potential bias of including FEP patients with less severe cognitive impairment. Nonetheless, several past cluster-analytic studies have also classified a large proportion of FEP or early psychosis patients (ranged: 43.9–62%) as cognitively-preserved subgroup (17, 21, 44). Fourth, age difference was found between cognitive subgroups, even though it was not independently predictive of cognitive cluster membership based on multivariable multinominal regression analyzes. This suggests that the effect of age on cognitive performance could be better accounted for in future studies on cognitive cluster analysis in early psychosis patients. Fifth, the cognitive assessment (alongside other study assessments) was undertaken when patients were clinically-stabilized with antipsychotic treatment, which may affect cognitive performance and confound the study results. Fifth, the relatively older mean age of our sample (age range of 26–55 years) may render our findings less comparable to the literature of first-episode research which mainly recruited younger patients with more typical age of onset (i.e., late adolescence or early adulthood) (69).

In conclusion, the current cluster analysis affirmed cognitive variability in a large cohort of adult FEP patients and identified three discrete cognitive subgroups with relatively-intact, intermediately-impaired (and mixed patterns of) and globally-impaired cognitive functioning. These cognitive subgroups were differentially associated with educational attainment, symptomatology, functional impairment and subjective QoL. Our findings thus suggest the potential utility of examining distinct cognitive subtypes to unravel their neurobiological underpinnings and genetic risk factors. Emerging data have in fact revealed that cognitive subgroups of schizophrenia are characterized by differences in neuroanatomical abnormalities (44, 70, 71). Additionally, our results underscore potential clinical implications of incorporating early identification of and provision of cognitive remediation (72, 73) to a subgroup of first-episode patients with global and severe cognitive impairment into the early psychosis service framework. This will facilitate improvement in cognitive deficits, psychosocial functioning and subjective QoL in first-episode patients during the early phase of illness.

## Data availability statement

The raw data supporting the conclusions of this article will be made available by the authors, without undue reservation.

## Ethics statement

The study involved human participants and was approved by the local institutional review boards, and written informed consent was obtained for all participants.

## Author contributions

EC designed the study. WC and CK conceptualized the research question and the analysis approach. CK performed statistical analyzes, interpreted the results and wrote the first draft of the manuscript. WC and VF interpreted the results, critically revised and finalized the manuscript. All authors contributed to the article and approved the submitted version.

## Funding

The study was supported the Hong Kong Jockey Club Charities Trust (21009144).

## Conflict of interest

The authors declare that the research was conducted in the absence of any commercial or financial relationships that could be construed as a potential conflict of interest.

## Publisher’s note

All claims expressed in this article are solely those of the authors and do not necessarily represent those of their affiliated organizations, or those of the publisher, the editors and the reviewers. Any product that may be evaluated in this article, or claim that may be made by its manufacturer, is not guaranteed or endorsed by the publisher.
