# Supplementary material for: Cognitive subgroups and the relationships with symptoms, psychosocial functioning and quality of life in first-episode non-affective psychosis: a cluster-analysis approach
Source: Front Psychiatry. 2023 Jul 27;14:1203655. doi: 10.3389/fpsyt.2023.1203655 (PMC10412814; doi:10.3389/fpsyt.2023.1203655)
Supplement: Supplementary file 1 [file Data_Sheet_1.docx]

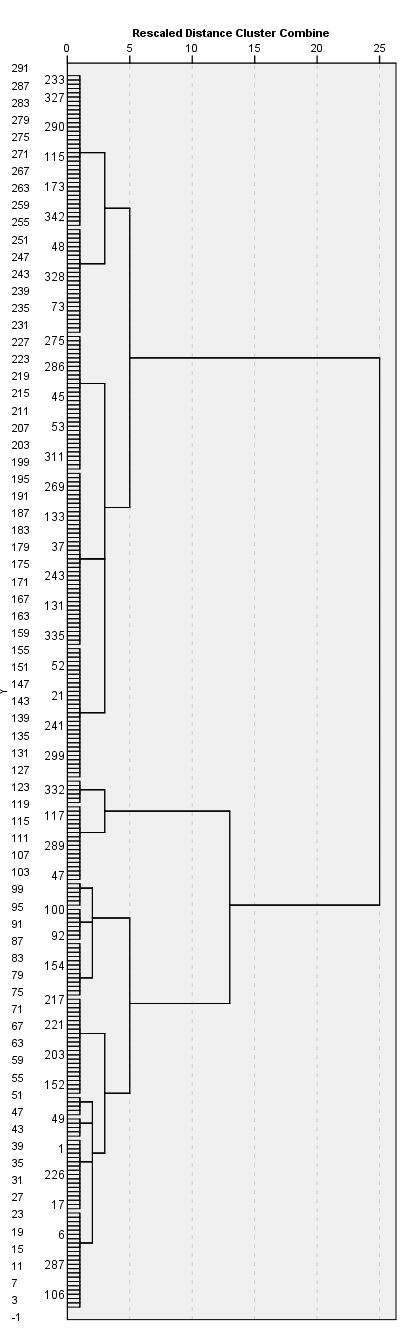
 (a)

(b)

**Supplementary Figure S1**. (a) Dendrogram and (b) Agglomeration scree plot of the hierarchical clustering on cognitive functioning of first episode psychosis patient sample.

**Supplementary Table S1.** Multivariate multinomial logistic regression analyses for patient characteristics (external variables) of cognitive clusters.

|  | GIC vs. RIC | | | GIC vs. IIC | | |
| --- | --- | --- | --- | --- | --- | --- |
| External variable | OR (95% CI) | *d* | *P* | OR (95% CI) | *d* | *P* |
| Age at entry | 1.01 (0.89-1.15) | 0.72 | 0.897 | 0.99 (0.89-1.10) | 0.39 | 0.842 |
| Years of education | 1.43 (1.25-1.63) | -1.37 | **<0.001** | 1.24 (1.11-1.38) | -0.66 | **<0.001** |
| Age at onset | 0.93 (0.82-1.04) | 0.66 | 0.208 | 0.99 (0.89-1.10) | 0.34 | 0.809 |
| Chlorpromazine equivalent dose | 1.00 (0.99-1.00) | 0.36 | 0.050 | 1.00 (1.00-1.00) | 0.24 | 0.139 |
| PANSS positive symptom score | 1.04 (0.92-1.18) | 0.39 | 0.499 | 0.97 (0.88-1.07) | 0.37 | 0.576 |
| PANSS disorganization score | 0.63 (0.49-0.81) | 0.89 | **<0.001** | 0.84 (0.74-0.96) | 0.54 | **0.011** |
| SANS total | 0.95 (0.89-1.01) | 0.17 | 0.092 | 0.98 (0.95-1.02) | 0.07 | 0.245 |
| Insight | 0.94 (0.69-1.30) | -0.05 | 0.718 | 0.95 (0.74-1.22) | 0.39 | 0.678 |
| SOFAS score | 1.01 (0.98-1.05) | -0.67 | 0.520 | 0.99 (0.96-1.02) | -0.25 | 0.470 |
| SF12 total score | 1.00 (0.99-1.01) | -0.38 | 0.795 | 1.01 (1.00-1.01) | -0.39 | 0.162 |

GIC, globally-impaired cognitive subgroup; IIC, intermediately-impaired cognitive subgroup; OR, odds ratio; PANSS, Positive and Negative Syndrome Scale;

RIC, relatively-intact cognitive subgroup; SANS, Scale for the Assessment of Negative Symptoms; SF12, 12-Item Short Form Survey; SOFAS, Social Occupational

Functioning Assessment Scale.
